# Understanding the Effect of Electron Irradiation on WS_2_ Nanotube Devices to Improve Prototyping Routines

**DOI:** 10.1021/acsaelm.4c01450

**Published:** 2024-12-13

**Authors:** Martin Kovařík, Daniel Citterberg, Estácio Paiva de Araújo, Tomáš Šikola, Miroslav Kolíbal

**Affiliations:** † CEITEC, Brno University of Technology, Purkyňova 123, 61200 Brno, Czech Republic; ‡ Faculty of Mechanical Engineering, Institute of Physical Engineering, Brno University of Technology, Technická 2896/2, 616 69 Brno, Czech Republic

**Keywords:** WS_2_, nanotubes, electrical properties, electron beam irradiation, prototyping

## Abstract

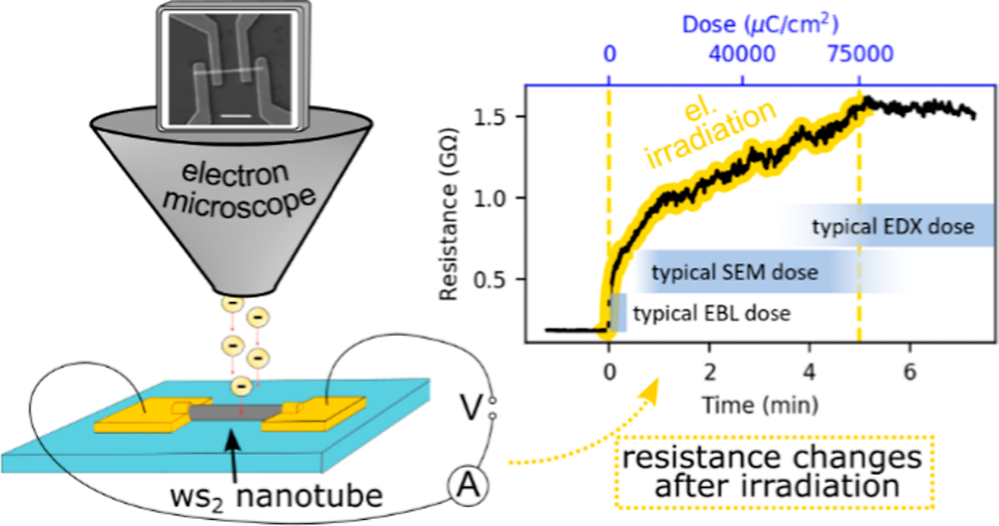

To satisfy the needs of the current technological world that demands high performance and efficiency, a deep understanding of the whole fabrication process of electronic devices based on low-dimensional materials is necessary for rapid prototyping of devices. The fabrication processes of such nanoscale devices often include exposure to an electron beam. A field effect transistor (FET) is a core device in current computation technology, and FET configuration is also commonly used for extraction of electronic properties of low-dimensional materials. In this experimental study, we analyze the effect of electron beam exposure on electrical properties of individual WS_2_ nanotubes in the FET configuration by in-operando transport measurements inside a scanning electron microscope. Upon exposure to the electron beam, we observed a significant change in the resistance of individual substrate-supported nanotubes (by a factor of 2 to 14) that was generally irreversible. The resistance of each nanotube did not return to its original state even after keeping it under ambient conditions for hours to days. Furthermore, we employed Kelvin probe force microscopy to monitor surface potential and identified that substrate charging is the primary cause of changes in nanotubes’ resistance. Hence, extra care should be taken when analyzing nanostructures in contact with insulating oxides that are subject to electron exposure during or after fabrication.

## Introduction

In the current world of rapidly evolving technology including areas such as the Internet of Things, artificial intelligence, or medical diagnostics, there is an increased demand for advanced electronic devices for computation or sensing. In order to satisfy these needs, the rapid development of new devices based on low-dimensional materials beyond silicon is required. New device concepts include, e.g., negative differential resistance FETs,^1^ reconfigurable nanowire-based Schottky barrier FETs^2,3^ etc. It is important to understand all of the processes during prototyping, including the role of electron beam exposure on material and device properties. Fabrication and analysis of electronic devices based on low-dimensional materials include the use of an electron beam (e-beam) in many cases. High-resolution photolithography, together with photomasks, is often inaccessible or too expensive for prototyping when only a small number of devices is made and the lithography design is frequently adjusted. Hence, electron beam lithography (EBL) is used for contact fabrication. In between the fabrication steps, the process is often checked with a scanning electron microscope. Some procedures require the use of energy-dispersive X-ray spectroscopy (EDX). However, it is well known that an e-beam can induce both reversible and irreversible changes in the nanomaterials under study or in the underlying substrate.^4−7^ Subsequently, the properties of the constituting material and thus of the device itself are generally altered. Therefore, understanding how electron irradiation affects properties of electronic devices under study is of crucial importance, especially in the case that the exposure by e-beam is an integral part of the process flow and cannot be avoided.

The effect of e-beam damage on materials has been widely studied by transmission electron microscopy,^8−10^ where high-energy e-beam (80–300 keV) is used to scan over selected nanoscale areas. On the contrary, EBL or SEM traditionally utilize lower electron energies. Common SEM observations are made utilizing electron energies in the range of 1–30 keV, and most laboratory EBL tools utilize 20–50 keV beams (although 100 keV tools have become more available recently). The previously established energy thresholds for knock-on damage in materials are usually above the low-energy range (e.g., 80 keV for C atoms in graphene^11^ or S atoms in MoS_2_^12^) and, hence, much less attention has been paid to tackle the damage induced by low-energy beams. Nevertheless, several recent reports have revealed a multitude of effects of a low-energy e-beam on properties of low-dimensional materials. These effects include the degradation of thin insulating gate oxide in metal–oxide–semiconductor field effect transistors (MOSFETs),^13,14^ the alteration of the optical properties of GaN,^15,16^ resist-free patterning, and phase transitions,^17,18^ or other significant changes in 2D material properties.^19−23^ The e-beam can influence materials even below the knock-on threshold via inelastic energy transfer^24^ (e.g., radiolysis) or secondary effects such as defect migrations, phase transformation, surface contamination, etc.^11,25^ These mechanisms have just begun to be discussed in the current literature.^26^

Instead of going into the very detail of the e-beam effect on certain materials, in this contribution, we aim to analyze the e-beam effect on a specific, but widely used scheme in low-dimensional materials research, namely, current–voltage (*IV*) characterization of 1D nanostructure in a field-effect-transistor (FET) configuration. Such measurements are routinely done to investigate electronic properties of materials in question, e.g., mobility extraction. Here, we have chosen WS_2_ nanotubes since the transition-metal dichalcogenide family in general is nowadays highly interesting for applications^2,3,27,28^ and, in particular, information about the effects of electron irradiation on WS_2_ nanotubes is very limited. Specifically for WS_2_ nanotubes, there are two papers on structural damage caused by high-energy electrons;^6,29^ however, consequences of electron irradiation with lower energies (1–30 kV) and, most importantly, with doses that are commonly used for sample observation or in lithography processes have not yet been systematically examined. We performed operando irradiation inside the SEM device combined with measurement of the *I*–*V* characteristics. In-operando electrical transport measurement during and after irradiation allowed us to directly observe the effect of the beam on electrical properties measured in the FET configuration in vacuum as well as to evaluate the effect of air exposure on the acquired nanotube characteristics after venting the microscope. Importantly, we employed in situ Kelvin probe force microscopy (KPFM) to provide information about changes in the work function and implanted charge.

The analyzed samples consisted of individual electrically contacted WS_2_ nanotubes on an oxide-passivated highly doped n-type silicon substrate. We used two different oxide insulating layers on top: 150 nm thermal SiO_2_ or 300 nm thermal SiO_2_ plus 25 nm HfO_2_ deposited by atomic layer deposition. Contacts to individual nanotubes were defined by EBL and fabricated by the evaporation of a Ti/Cu/Au layered stack with thicknesses of 5/300/20 nm. Then the samples were wire-bonded to a chip expander with contact pins to allow a wired connection for electrical measurement inside the SEM chamber. *I*–*V* characterization was performed in a 4-probe setup to separate the resistance of contacts and the nanotube. In total, 9 nanotubes were analyzed in detail during electron irradiation.

## Results and Discussion

**Figure 1:**
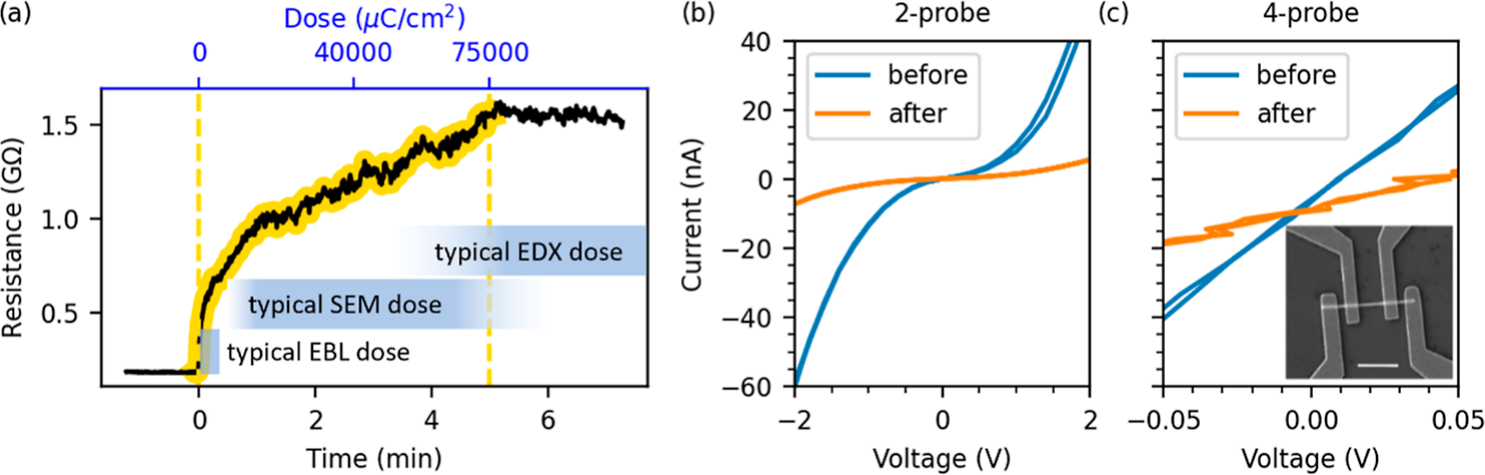
(a) Time evolution of WS_2_ nanotube resistance during electron irradiation (75 mC/cm^2^, 30 keV electrons, indicated by a yellow background and yellow dashed lines). The inset bars compare the dose used in this experiment (top axis) to typical doses commonly used in EBL, SEM imaging, and EDX analysis. (b) 2-Probe *I*–*V* curves obtained before and after irradiation. (c) 4-Probe *I*–*V* curves obtained before and after irradiation. The inset shows a typical SEM image of a contacted nanotube; the scale bar is 2 μm.

Our first key observation is that the electrical properties of WS_2_ nanotubes are changed significantly after the irradiation (30 kV, 250 pA, scanning 10 × 10 μm^2^ area of the substrate with nanotubes above, which corresponds to a fluence of 250 μC·cm^–2^·s^–1^). The original resistance varied substantially between individual nanotubes, typically ranging from several MΩ to a few GΩ. Nevertheless, after 5 min of irradiation, the resistance of all nanotubes increased by a factor of 2 to 14. All measured nanotubes showed a rapid increase in resistance at the beginning of the exposure (doses up to several tens of mC/cm^2^), and then slowly reaching a nearly saturated value (Figure 1a). For comparison, typical doses for SEM imaging, EBL, and EDX are noted in Figure 1a; our results confirm that even SEM imaging is sufficient to change the measured electrical properties significantly. EDX chemical analysis would be even more detrimental as it uses higher currents and acquisition times. If we compare the results with doses typically used in EBL with PMMA resists (100–1000 μC/cm^2^), it would be equivalent to a few seconds of our standard SEM irradiation; therefore, the effect of EBL would be weaker than the total change observed although still considerable.

Figure 1b,c shows the *I*–*V* curves measured before and after irradiation in the 2-probe and 4-probe configurations, respectively. The 2-probe *I*–*V* characteristic shows high asymmetry caused by Schottky barriers at the metal–semiconductor interface, whereas the 4-probe *I*–*V* measurement has linear ohmic behavior, highlighting the importance of the 4-probe technique for correct characterization of the nanotube. Nevertheless, both techniques indicate a significant increase of resistance after irradiation by 75 mC/cm^2^ with 30 keV electrons. The increase of device resistance by 1 order of magnitude can, in general, significantly affect parameter extraction, such as Hall mobility, which would be underestimated by the same factor.

**Figure 2:**
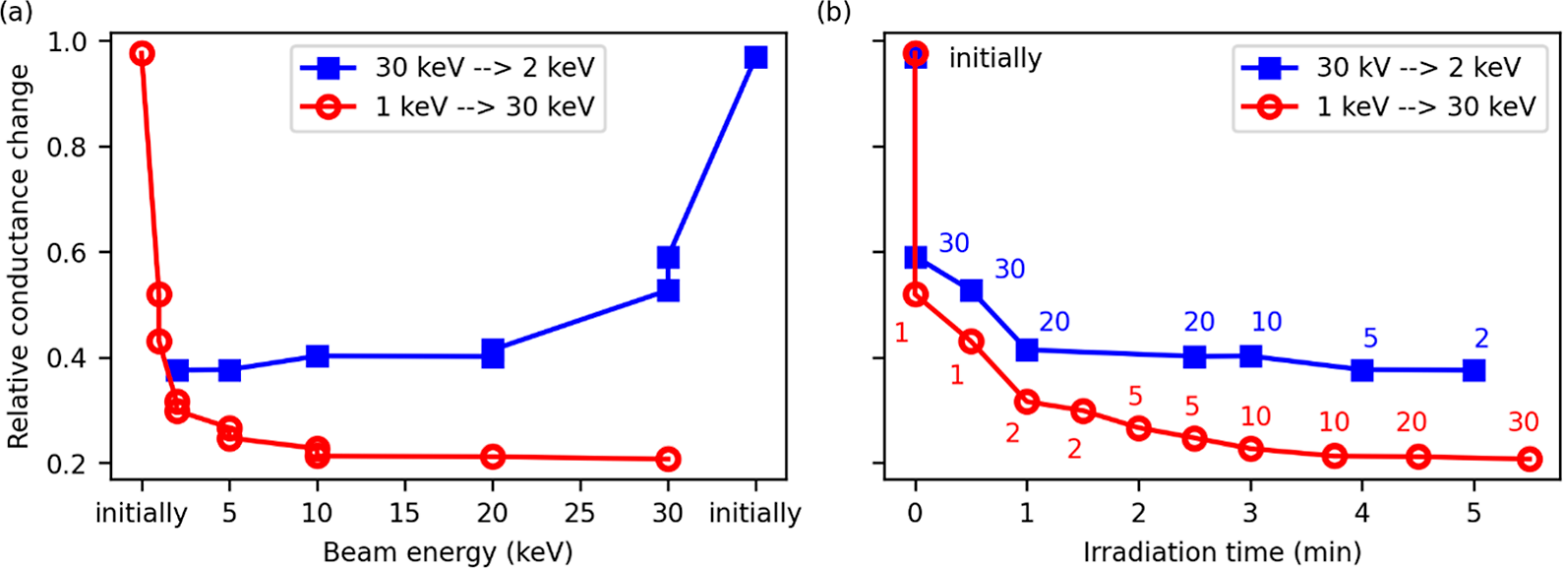
Effect of electron beam energy on the conductance of the WS_2_ nanotubes. (a) Two different nanotubes were irradiated for a defined time with various beam energies ranging from 1 to 30 keV. The irradiation of one nanotube began at a low beam energy of 1 keV, gradually increasing in steps to a maximum of 30 keV (red). The other nanotube was irradiated in reverse order, starting at 30 keV and changing to 2 keV (blue). (b) The same measured conductance values plotted as a function of the irradiation time. There is a correlation between the conductance change and irradiation time rather than the acceleration voltage, which means that the conductance change depends mainly on the total irradiation time, not the beam energy. The numbers correspond to the actual acceleration voltage that was used for each point.

We also examined the influence of the electron beam energy on resistance changes and found no significant dependence. Note that for better clarity, the figures actually show conductance (inverse value of resistance). The absence of a distinct response to different acceleration voltages (Figure 2a) confirms the absence of knock-on damage in the nanotube itself; instead, different mechanisms are in play, and we will discuss this issue further. Data in Figure 2b show that the major parameter affecting the measured physical properties is the total irradiation time (dose). For this reason, we carried out all the irradiation experiments with a 30 kV beam and 250 pA, which is the common setting for EBL and is relevant to routine SEM imaging as well.

**Figure 3:**
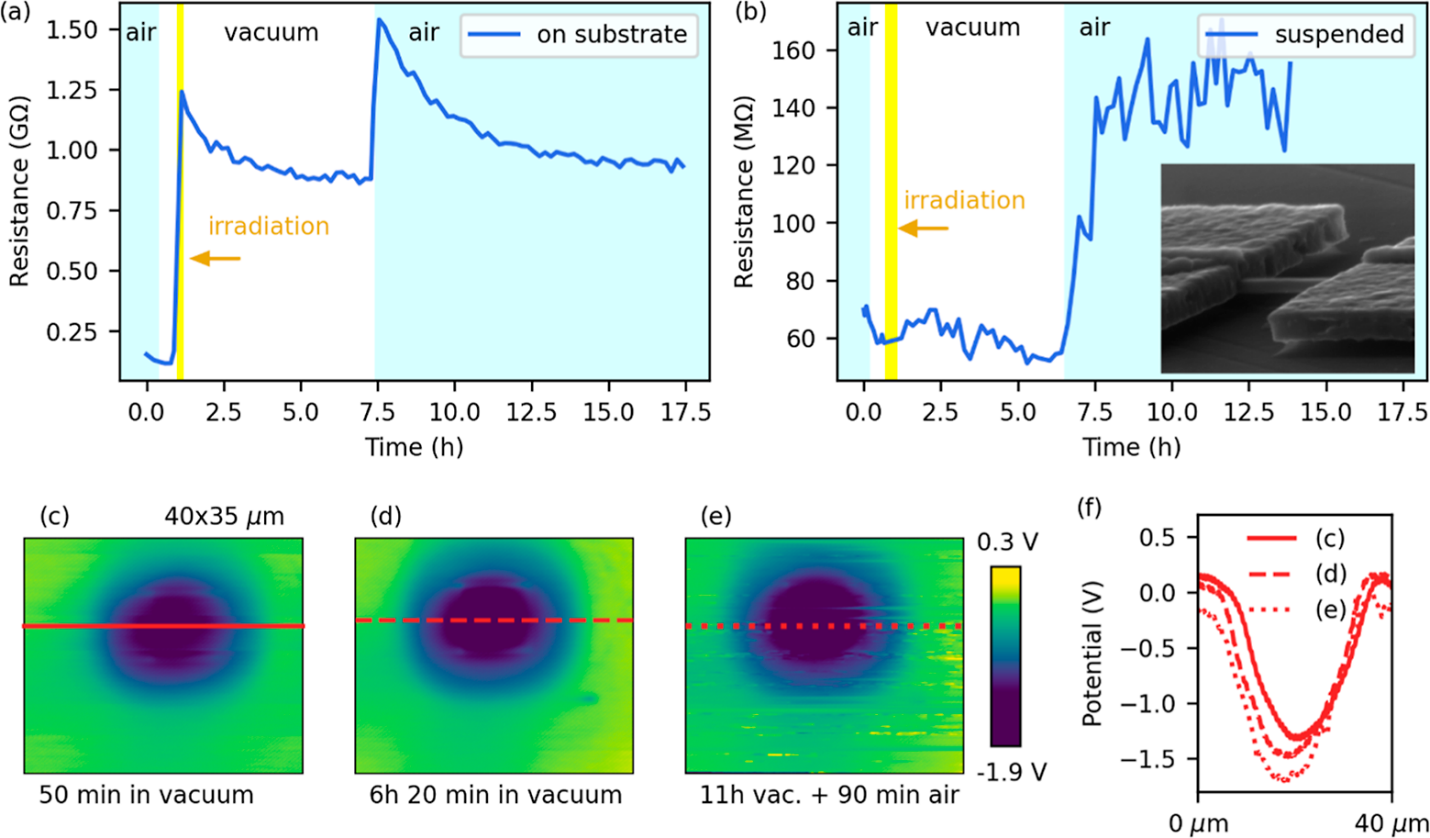
(a) Evolution of the 2-probe resistance of a typical WS_2_ nanotube on a substrate with an insulating HfO_2_ layer throughout the whole experiment. Electron irradiation causes a long-term resistance increase that persists both in vacuum and in ambient conditions. (b) Evolution of the two-probe resistance of a suspended WS_2_ nanotube shows a minimum response to the e-beam compared to the nanotube on the substrate (supported), highlighting the role of the substrate. The inset shows the SEM image of a suspended nanotube. (c–e) Surface potential images of the substrate used in (a) after electron irradiation measured by KPFM. Electron irradiation leads to substrate charging, which remains present in both vacuum and ambient conditions. All KPFM images have the same color range, and the potential profiles across the lines in the images can be seen in (f).

Next, we aim to clarify how the nanotube resistance evolves over time after it has been exposed to a controlled dose by the e-beam. We measured the *I*–*V* curves of the nanotube every 10 min for over 15 h after irradiation had finished, both in vacuum and in air. The data obtained by the 2-probe measurement is shown in Figure 3a. The data from the 4-probe configuration is provided in the Supporting Information, Figure S1a. The observed resistance change in time consists of two parts: first, it decays slowly after irradiation (short-term resistance change), yet it does not return to the initial value even after tens of hours (long-term resistance change). The time constant of the initial decrease in vacuum, obtained by fitting an exponential, ranges between 1 and 5 h. Exposure to air did not restore the resistance to its preirradiation value. Instead, the resistance increased again, likely due to molecular (water, oxygen, ...) adsorption on the surface.^30^ This is consistent with preirradiation measurements in both air and vacuum, where resistance is also higher in air (Supporting Information, Figure S2).

In the following text, we examine and discuss the origin of this behavior. Since the substrate clearly plays a major role in conductivity changes, we first focus on substrate charging and the resulting field effect. Next, we briefly discuss the changes induced in the nanotube itself (structural damage, electronic transitions, charge trapping), modification of the nanotube surface (contamination build-up), and modification of contacts.

To assess the impact of substrate charging, we also analyzed suspended nanotubes that are in contact with the substrate only at their ends. The fabrication process included immersing the sample in concentrated hydrofluoric acid for 8 s to etch the substrate oxide beneath the nanotubes after fabricating the contacts. Using SEM and Raman spectroscopy, we verified that the nanotubes remained undamaged by the etching procedure (inset of Figure 3b, Supporting Information, Figure S3). Our results in Figure 3b show that the resistance change of suspended nanotubes after electron irradiation is negligible compared to the supported nanotubes (lying on the substrate).

To decipher the role of the substrate in the resistivity change of nanotubes, we used in situ KPFM, an AFM-related technique that utilizes a sharp conductive tip for measurement of surface electrical potential.^31^ In this way, it is possible to detect a local charge trapped in the substrate oxide that can potentially explain the conductivity change by inducing an undesired field-like effect. We performed an experiment analogous to the irradiation of the WS_2_ nanotubes. We exposed a 10 × 10 μm^2^ area of a substrate without a nanotube (30 kV, 250 pA, 5 min) and used KPFM to image charge build-up at an equivalent time scale after irradiation. Selected KPFM images (Figure 3c–e) clearly show an implanted charge within the irradiated area and its development over time under both vacuum and then ambient conditions; Figure 3f shows the potential profiles across the lines marked in the KPFM images. Before we discuss this observation, we want to make sure our findings have general validity and are not caused by a specific nanotube–substrate interaction. We performed the same experiment on two substrates with different gate oxides—ALD-deposited HfO_2_ and thermal SiO_2_. On both substrates, we observed qualitatively the same electrical transport results and the KPFM substrate charging as well (here we present only the results on HfO_2_ substrates; the results on SiO_2_ can be viewed in Supporting Information, Figure S4).

Based on the KPFM potential measurements and considering our specific KPFM setup (sample biased, tip grounded), we can infer that the irradiated area is positively charged as more electrons are ejected than captured. The KPFM images also show no significant discharging of the substrate over time, both in vacuum and air (Figure 3c–f). This suggests a relationship between long-term resistance changes and substrate charging. The positive charge accumulated in the substrate repels the holes in the p-type nanotube, increasing its resistance due to a reduced majority carrier density. These findings are consistent with our 4-probe and FET transport measurements (Supporting Information, Figures S5 and S6).

The effect of the substrate is further confirmed by the observation that resistance changes occur only in supported nanotubes and not in suspended ones (Figure 3a,b). However, the short-term resistance change in supported nanotubes, characterized by time constants of a few hours, remains to be explained. Generally, charge trapped at the 1D nanostructure surface or in surface oxide can have long lifetimes and induce transient behavior with time constants of several hours, which has been already observed for Ge nanowires.^32,33^ However, suspended nanotubes did not exhibit this behavior. In addition, oxidation of the nanotube surface due to irradiation is not expected.^34^ Hence, the short-term resistance change is also related to the substrate. The characteristic time constants are within the ranges reported in charge diffusion experiments^35^ and those related to slow charge trapping in defective oxides.^36^ Moreover, Burson et al.^37^ monitored charged impurity density on a SiO_2_ substrate after electron exposure and observed almost identical time dependence as the nanotube resistance in our experiments in Figure 3a, reporting a time constant of 10 h. Different charge redistribution in the oxide can lead to changes in the device’s electronic properties.^38^ Therefore, the short-term resistance change is caused by the charge trapped in the substrate oxide in the nearest vicinity of the nanotube that is being slowly detrapped.

The effect of substrate charging can be mitigated by postexposure annealing. In our case, the common detrapping procedure of mild annealing in ambient conditions for 30 min at 90 °C partially removed the trapped charge (Supporting Information, Figure S7). For complete detrapping, longer annealing at higher temperatures is needed. For example, annealing at 250–300 °C for 30 min should be sufficient to remove the trapped charge from SiO_2_.^37^ The observed detrapping is a strong argument against permanent structural damage to the gate oxide by the electron beam. Considerable beam structural damage would be expected at higher accelerating voltages (300 kV) and significantly higher doses (8 C/cm^2^).^5^

The electron beam can potentially generate structural defects in the WS_2_ nanotubes as well. The most common defect in WS_2_ induced by electron irradiation is the formation of sulfur vacancies.^25,39^ The theoretical electron knock-on damage threshold for WS_2_ is between 70 and 100 keV,^12,40^ far above the beam energy we used. However, it has been observed that creation of sulfur vacancies is also possible for lower acceleration voltages of 30 kV and below.^24,41^ Therefore, in order to evaluate the possibility of a structural damage formation, we have chosen Raman spectroscopy as it is capable of detecting sulfur vacancies in TMDs (although with limited sensitivity^42^) and at the same time is nondestructive at a proper excitation intensity and allows single nanotube analysis. The Raman analysis (see the Supporting Information, Figure S8) suggests there is no significant structural damage to the nanotubes. We did not observe any shifts in the position of spectral components that could be correlated to generation of sulfur vacancies.^42^ We observed small changes in the component ratios; however, there was no trend that could be correlated to the increase of nanotubes’ resistance after irradiation. At this point, it is important to note that structural damage is usually detected if much higher doses are used, as summarized in Table 1. Furthermore, de Graaf reported that number of vacancies generated in WS_2_ upon the e-beam irradiation is independent of the e-beam current density used, as long as the total exposure dose is the same,^41^ validating the use of the total dose as a quantifiable measure of defect generation. Altogether, the doses used in our study, which are relevant to common SEM observations and EBL, are not large enough to generate electronically valid structural defects.

**Table 1:** Effect of Irradiation on WS_2_ Reported in Other Previous Studies^a^

WS_2_ form	beam energy (keV)	dose (μC/cm^2^)	effect	source
2D FET	1	1.8	FET threshold voltage shift	(20)
nanotubes	30	10^2^ to 10^5^	resistance increase	this work
2D	30	2.4 × 10^7^	vacancies move	(43)
nanotubes	200	2.6 × 10^7^	recovery of damage caused by mechanical bending	(44)
2D	80	2.7 × 10^8^	resistance increase	(45)
2D (1L)	60	1.6 × 10^8^	phase transformations	(46)
2D	30	4.0 × 10^8^	vacancy generation	(41)
2D	100	3.0 × 10^12^	vacancy generation	(47)
nanotubes	200	>10^11^	critical damage	(6)

a The order is with respect to the total dose used for irradiation.

Prolonged exposure to e-beam can result in contamination build-up (especially in supported nanotubes) and a related surface-induced charge carrier modulation.^48^ Here, we have not detected any carbon contamination by Raman^49^ (Supporting Information, Figure S9). Moreover, both suspended and supported nanotubes remain sensitive to air exposure, indicating that there is no thick contamination layer.

Finally, we also tested the effect of the e-beam on contacts by irradiating individual parts of the nanotube separately (see the Supporting Information, Figure S10). The change in conductance occurs after e-beam exposure of both the nanotube itself and the contacts. This observation is in agreement with our explanations that the substrate charge-induced field effect influences the local Fermi level position and consequently also the Schottky barrier height at the place of the contact.

## Conclusions

In summary, we observed that exposure of nanotube devices in FET configuration to a low-energy electron beam with conditions comparable to standard SEM observations (1–30 kV, 250 pA, 1–300 s) significantly changes the electrical properties of substrate-supported nanotubes, namely, their resistance. The resistance extracted from FET measurements is a key quantity for determination of critical electronic properties, e.g., charge carrier mobility, which can thus be largely affected by the prototyping process and postfabrication inspection. Importantly, the resistance does not return to the original value, even after several days. Our results also show that varying the beam energy in the range of 1 to 30 keV has a negligible effect on the resistance change; the key factor is the total dose. By analyzing suspended nanotubes as well as employing in situ KPFM, we have found that the origin of this behavior is the field effect induced by charges generated in the gate oxide. Our study highlights the importance of understanding the impact of electron exposure on supported 1D nanodevices as it is commonly used during both their fabrication and analysis. This effect is likely to be even more pronounced in 2D materials, where the field effect is more effective in modulating the electronic properties. Therefore, our conclusions stress the importance of utilizing suspended geometry for the correct extraction of electronic properties of low-dimensional materials and add further key findings to the ongoing debate.

## Methods

### Fabrication of the Device

Si wafers with 150 and 300 nm thermal SiO_2_ were purchased from Siegert Wafer and onsemi, respectively. An additional HfO_2_ layer was deposited by a standard atomic layer deposition process in an Ultratech Fiji reactor. WS_2_ nanotubes were fabricated by high-temperature sulfurization in a fluidized bed reactor, as described elsewhere.^50,51^

Powder with WS_2_ nanotubes was dispersed in isopropyl alcohol by sonication and then drop-cast onto a substrate with prefabricated alignment marks. Both the alignment marks and the contacts to the nanotube were defined by EBL (Tescan MIRA3 SEM + RAITH Elphy lithography module) using a PMMA resist. Then, contacts were fabricated by evaporation of a Ti/Cu/Au layered stack with thicknesses of 5/300/20 nm (electron beam evaporator BESTEC).

### Electrical Characterization

Samples with contacted nanotubes were wire-bonded to a chip expander (Seant Technology) with contact pins to allow a wired connection for electrical measurement inside the scanning electron microscopy (SEM) chamber. *I*–*V* transport characterization was performed with a CascadeMicrotech MPS-150 probe station coupled with a Keithley S4200 (after fabrication under ambient conditions) or with a Keithley 2636B (experiments inside the SEM instrument). Both setups allowed 4-probe measurements to separate the resistance of contacts and the nanotube. Electron irradiation was performed in a Tescan LYRA3 SEM with a base pressure of 10^–4^ Pa.

### In Situ Kelvin Probe Force Microscopy

In situ KPFM measurements were carried out inside the SEM chamber immediately after electron irradiation using a NenoVision Litescope atomic force microscope. The KPFM was operated in a frequency-modulated regime^52^ as it is less prone to stray capacitance artifacts and therefore provides more accurate surface potential values compared to more common amplitude-modulated KPFM. In our setup, the sample was grounded and the tip biased, resulting in the positive substrate charge appearing as a lower surface potential in the KPFM images.

### Raman Spectroscopy

The Raman spectroscopy measurements were performed with a WiTec Alpha 300R using a 532 nm excitation laser with a power of 0.2 mV.

## Data Availability

The data that support the findings of this study are available from the corresponding author upon reasonable request.

## Supporting Information Available

Raman spectroscopy of e-beam-irradiated WS_2_ nanotubes, additional transport measurement of WS_2_ nanotubes, and KPFM measurements of the irradiated SiO_2_ substrate (PDF)

## Abbreviations

AFM: atomic force microscopy
EBL: electron beam lithography
FET: field effect transistor
KPFM: Kelvin probe force microscopy
SEM: scanning electron microscopy
