# Supplementary material for: Understanding the Effect of Electron Irradiation on WS2 Nanotube Devices to Improve Prototyping Routines
Source: ACS Appl Electron Mater. 2024 Dec 13;6(12):8776–82. doi: 10.1021/acsaelm.4c01450 (PMC11673106; doi:10.1021/acsaelm.4c01450)
Supplement: Supplementary file 1 — el4c01450_si_001.pdf [file el4c01450_si_001.pdf]

Supporting Information for the article

Understanding the effect of electron irradiation on WS_2 nanotube devices to improve prototyping routines

Martin Kovařík[1,*], Daniel Citterberg[1], Estácio Paiva de Araújo[1], Tomáš Šikola[1,2] and Miroslav Kolíbal[1,2]

[1]*Brno University of Technology, CEITEC, Purkyňova 123, 61200 Brno, Czech Republic*

[2]*Brno University of Technology, Faculty of Mechanical Engineering, Institute of Physical Engineering, Technická 2896/2, 616 69 Brno, Czech Republic*

martin.kovarik@ceitec.vutbr.cz

Fig. S1: Effect of electron irradiation on WS_2 nanotube on a HfO2 substrate S-2

Fig. S2: I-V curves of WS2 nanotube measured in air and in vacuum S-2

Fig. S3: Raman spectra of pristine and HF treated nanotubes S-3

Fig. S4: Effect of electron irradiation on a WS2 nanotube on the SiO2 substrate S-4

Fig. S5: I-V characteristics of individual parts of the contacted nanotube S-5

Fig. S6: 4-probe I-V characteristics of a nanotube for different back-gate voltages and under light illumination S-5

Fig. S7: Effect of electron irradiation on surface potential of HfO2 substrate S-6

Fig. S8: Raman spectra of pristine and irradiated nanotubes S-7

Fig. S9: Raman spectra of the nanotubes and the substrate in range of 1000-4000 cm^{-1} to see potential carbon contamination S-8

Fig. S10: Conductance temporal development when different parts of a nanotube were irradiated S-9

References S-10

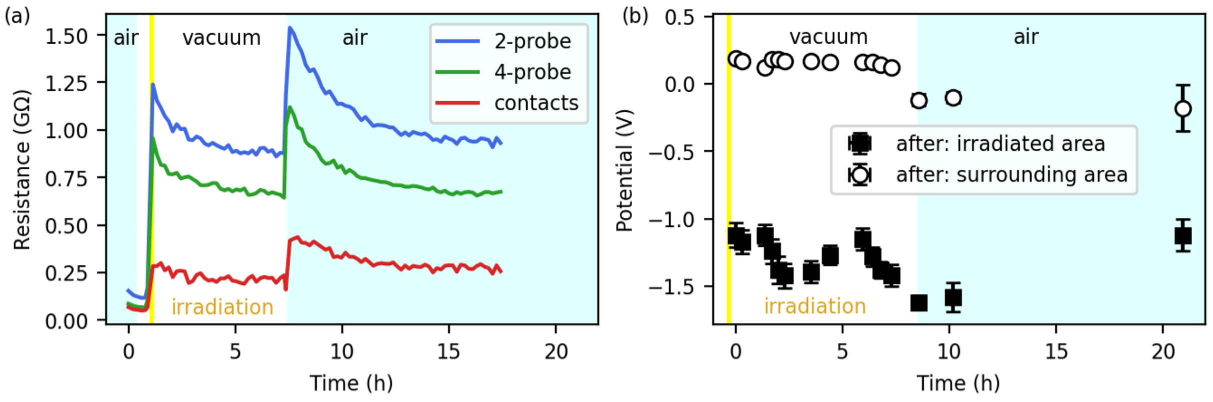

Fig S1: Effect of electron irradiation on WS_2 nanotube on a HfO_2 substrate. A nanotube was irradiated in SEM and its resistance measured during the whole experiment– in air, in SEM vacuum before and after irradiation, then again in air after venting the microscope in 10 minute intervals. The resistance of contacts was obtained by subtraction of the 2-probe resistance and the 4-probe resistance. (a) Nanotube resistance measured over time. (b) Surface potential of the SiO_2 substrate after irradiation. Black squares correspond to the surface potential measured directly at the irradiated area and circles to the surrounding measured area that was not irradiated.

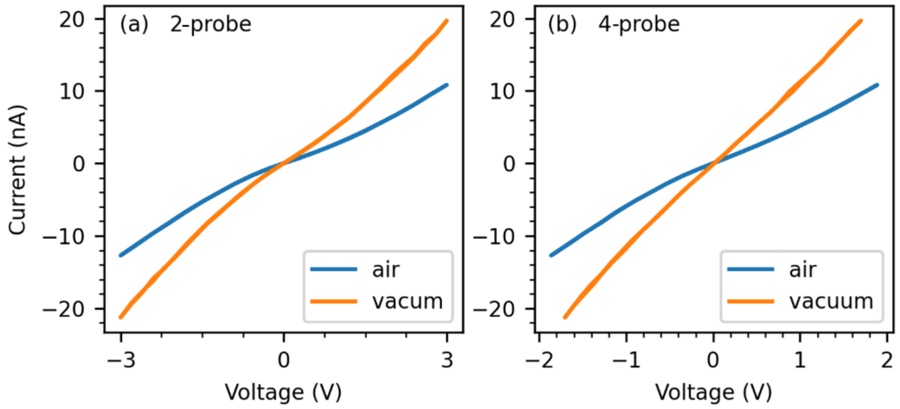

Fig S2: I-V curves of WS_2 nanotube measured in air and in vacuum with (a) 2-probe and (b) 4-probe technique. In vacuum, molecules from the nanotube surface desorb which leads to lower resistance. This trend was consistent throughout all analyzed nanotubes.

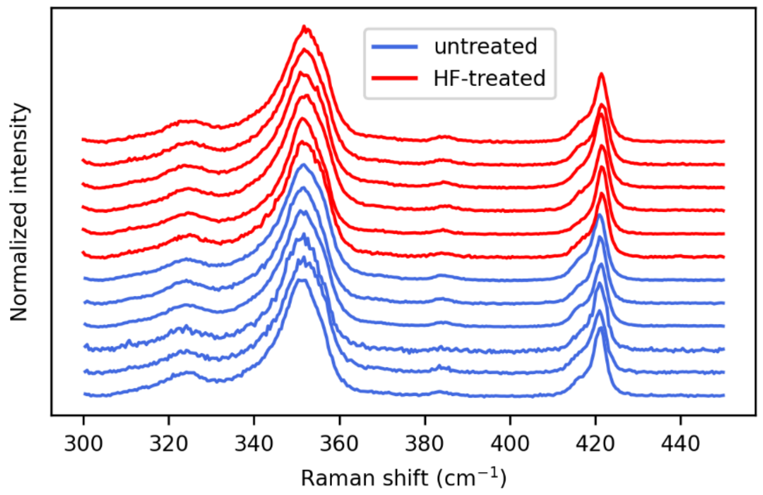

Fig S3: Raman spectra of six different untreated nanotubes (blue) and six HF-treated nanotubes (red) confirms that the nanotubes were not damaged by HF etching. The excitation wavelength was 532 nm, laser power 0.2 mW.

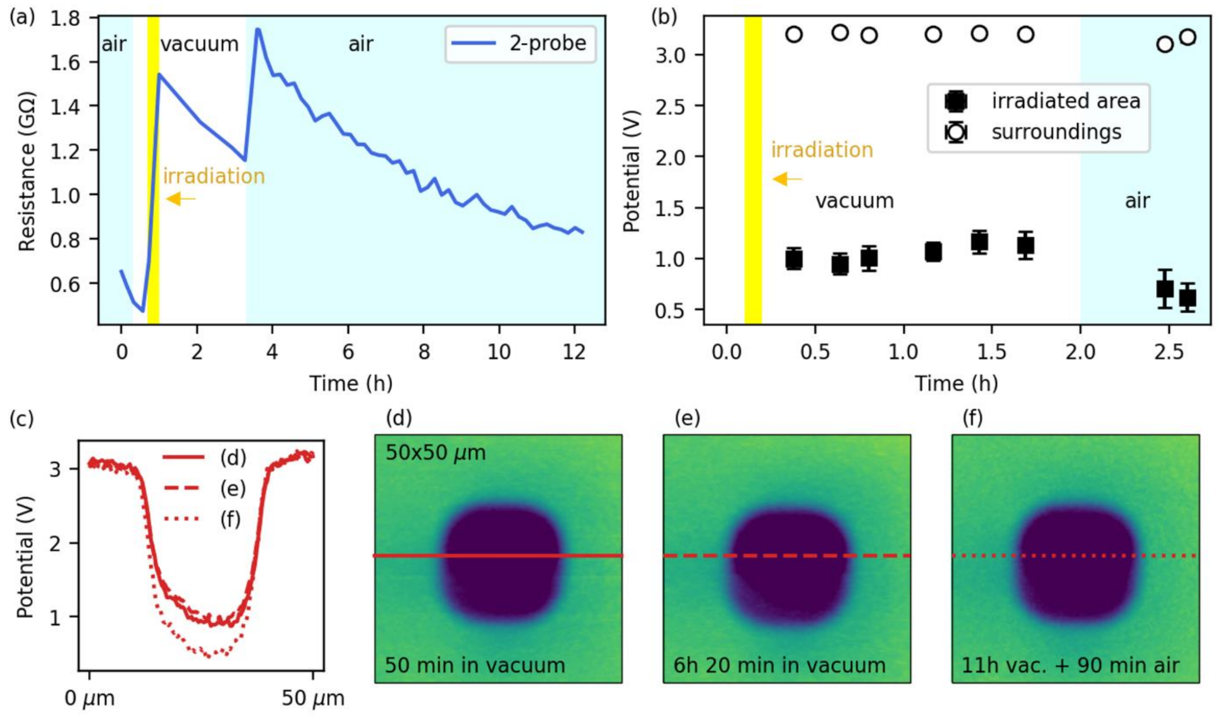

Fig S4: Effect of electron irradiation on a WS_2 nanotube on the SiO_2 substrate. The nanotube was irradiated in SEM and its resistance measured during the whole experiment – in air, in SEM vacuum before and after irradiation, then again in air after venting the microscope. (a) Nanotube resistance measured over time. (b) Surface potential of the SiO_2 substrate after irradiation. The black squares correspond to the surface potential directly at the irradiated area and the circles to the surrounding measured area that was not irradiated. (c) Potential profiles across the lines marked in the KPFM images (d), (e) and (f) of the irradiated substrate area corresponding to different times after substrate exposure to the electron beam. The KPFM images prove the substrate is charged by the electron beam. SEM irradiation parameters were exactly the same for the nanotube and substrate exposure: Scanning 10x10 µm, 30 kV, 250 pA.

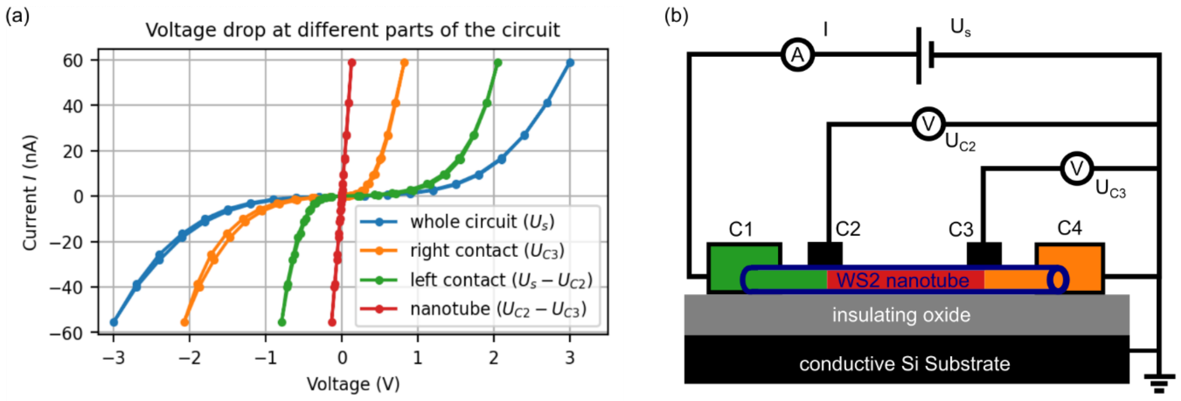

Fig S5: (a) I-V characteristics of individual parts of the contacted nanotube as shown in (b). Source voltage U_s was applied between outer contacts C1 and C4 and current I was measured. Simultaneously, voltage U_{C2} and U_{C3} at contacts C2 and C3 was measured as well. Therefore, we could plot the I-V characteristics of individual parts of the nanotube in (a): the whole circuit including the nanotube and contacts (blue), contact-nanotube interface at contacts C1 and C4 (green and orange, respectively) and the nanotube itself (red). The I-V curves at contacts C1 and C4 shows typical Schottky asymmetric behavior of metal-semiconductor junction and the contacts act as back-to-back Schottky diodes. It can be seen that for the negative source voltage, the junction at C1 is forward biased whereas at C4 reverse biased (for the positive source voltage vice versa). This leads us to the conclusion that the nanotube is the p-type, as for this doping type, the metal-semiconductor junction is forward biased when there is the positive bias on the semiconductor.

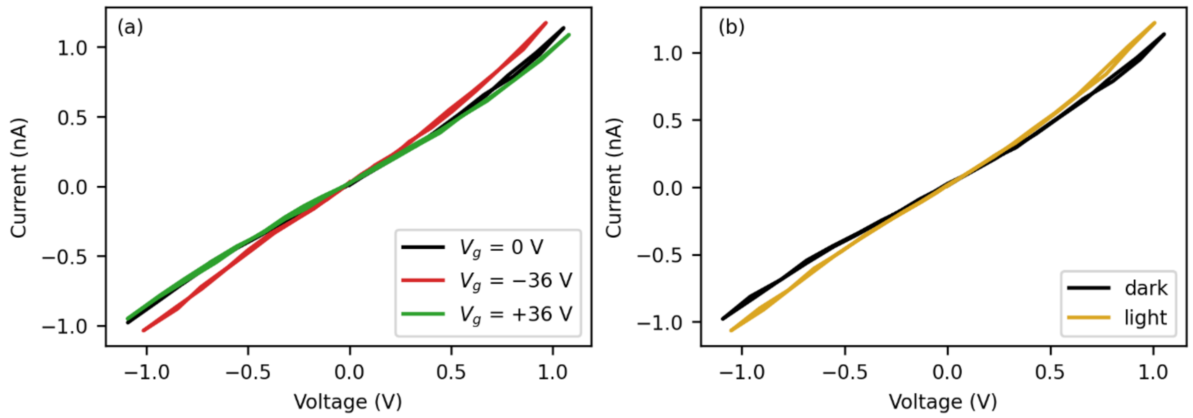

Fig S6: (a) Typical 4-probe I-V characteristics of a nanotube for different back-gate voltages. The nanotubes generally showed a very weak back-gate dependence with slightly increased conductivity for the negative back-gate bias (- 36 V). This confirms the p-type nature of the nanotubes. (b) Typical 4-probe I-V characteristics of a nanotube measured in the dark and under white light illumination. We can see that the effect of light illumination is much weaker than the observed changes after electron irradiation.

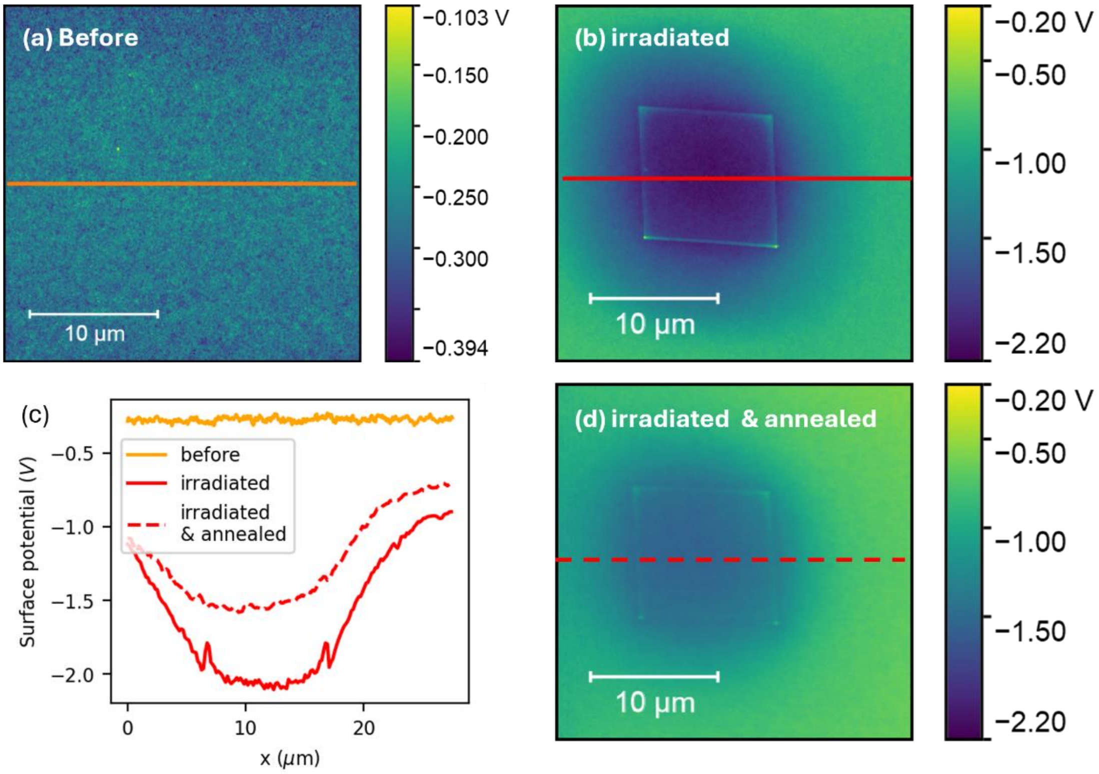

Fig. S7: Surface potential of $HfO_2/SiO_2/Si$ substrate (a) before electron irradiation, (b) after electron irradiation, (d) after annealing for 30 min at 90 °C and (c) line profiles from all images at marked positions. Annealing clearly shows partial reduction of the surface potential dip, which corresponds to partial release of the trapped charge. Irradiation parameters: 30 kV, 250 pA, 5 min, 10x10 µm square.

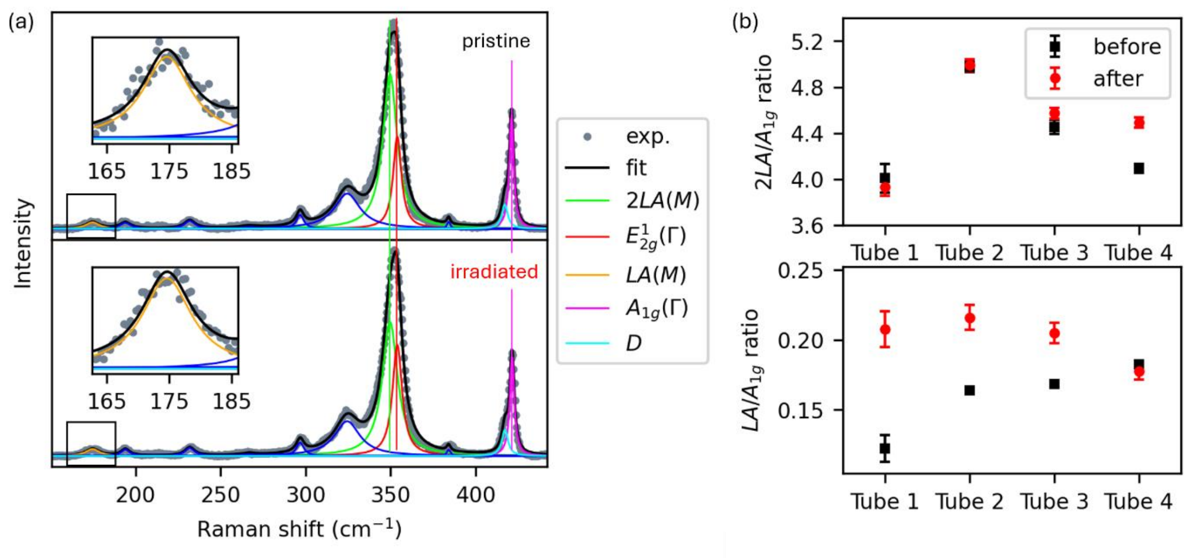

Fig. S8: (a) Raman spectra of 150-450 cm^{-1}, including deconvolution into individual peak components: before (top) and after (bottom) electron irradiation. The excitation wavelength was 532 nm, laser power 0.2 mW. The polarization of incident light with respect to the nanotube orientation was kept the same for measurement before and after irradiation. We did not observe any peak position shift as would be expected if sulfur vacancies were created [1]. In addition to the commonly observed components (E2g, A1g, LA, 2LA), there is additional component at A1g position, sometimes labelled as D, which could be a result of curvature-induced strain [2, 3] in the WS_2 layers or due to presence of sulfur vacancies in nanotube [4, 5, 6]. (b) The ratio of 2LA to A1g (top) and LA/A1g (bottom) component integral area before and after irradiation. The ratio of A1g to LA or 2LA is commonly used for characterization of defects in TMDS [7, 8]. LA/A1g ratio shows a moderate increase after irradiation. However, the absolute LA peak intensity is quite small, carrying a small signal-to-noise ratio. 2LA/A1g ratio is less prone to noise-induced uncertainties as the 2LA mode has a higher intensity. There is no clear trend visible in the data. In addition, the absolute values of LA/A1g ratios we report are significantly lower to other studies [8], suggesting very low defect concentration. Hence, it is plausible to state that our data show no trend that would suggest systematic and significant generation of defects in WS_2 by the irradiation.

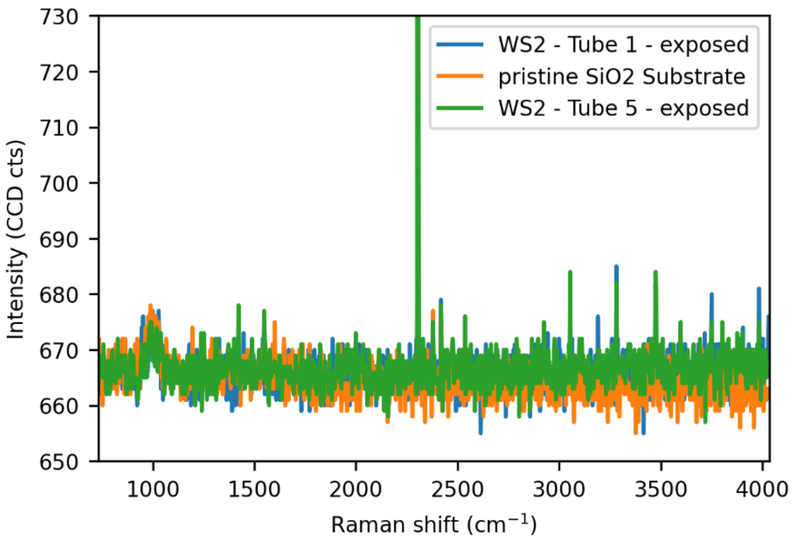

Fig S9: Raman spectra of two electron-irradiated nanotubes on SiO_2 and plain nonirradiated SiO_2 substrate. Carbon contamination is expected to cause peaks at approx. 1400 cm^{-1} and 1600 cm^{-1}, but no such peaks are detected. Therefore, the amount of carbon deposited by electron irradiation is probably very small. Excitation wavelength was 532 nm, laser power 0.2 mW.

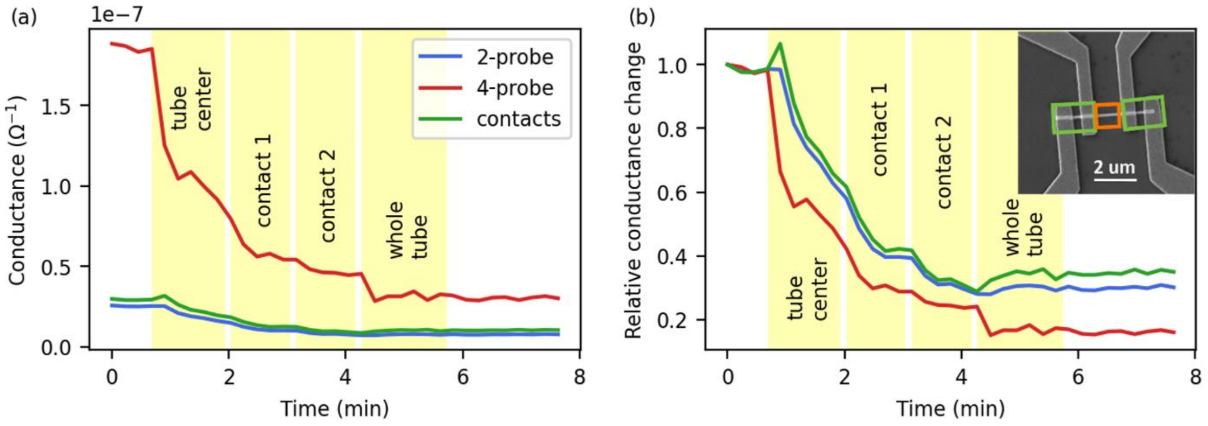

Fig S10: Conductance temporal development when different parts of a nanotube were irradiated – first nanotube center without contacts, then left and right contacts separately (see the inset panel b) and finally the whole nanotube together with contacts. The measurements were done simultaneously with the 2-probe and 4-probe technique. Resistance of contacts was calculated as a difference between the 2-probe and the 4-probe measurements. (a) Absolute conductance values, (b) normalized values to better visualize the relative conductance change. The inset of (b) shows the areas on the nanotube that were separately irradiated.

References

1. Parkin, W. M., Balan, A., Liang, L., Das, P. M., Lamparski, M., Naylor, C. H., Rodríguez-Manzo, J. A., Johnson, A. T. C., Meunier, V., Drndić, M. Raman Shifts in Electron-Irradiated Monolayer MoS_2. *ACS Nano* **2016**, 10, 4134–4142. DOI: 10.1021/acsnano.5b07388.
2. Krause, M., Virsek, M., Remškar, M., Kolitsch, A., Möller, W. Diameter Dependent Raman Scattering of WS_2 Nanotubes. *Phys. Status Solidi B* **2009**, 246, 2786–2789. DOI: 10.1002/pssb.200982275.
3. Velický, M., Rodriguez, A., Bouša, M., Krayev, A. V., Vondráček, M., Honolka, J., Ahmadi, M., Donnelly, G. E., Huang, F., Abruña, H. D., Novoselov, K. S., Frank, O. *The Journal of Physical Chemistry Letters* **2020**, 11, 6112–6118. DOI: 10.1021/acs.jpclett.0c01287
4. Lee, C., Jeong, B. G., Kim, S. H., et al. Investigating Heterogeneous Defects in Single-Crystalline WS_2 via Tip-Enhanced Raman Spectroscopy. *npj 2D Mater Appl* **2022**, 6, 67. DOI: 10.1038/s41699-022-00334-4
5. Lee, C., Jeong, B. G., Yun, S. J., Lee, Y. H., Lee, S. M., Jeong, M. S. Unveiling Defect-Related Raman Mode of Monolayer WS_2 via Tip-Enhanced Resonance Raman Scattering. ACS Nano 2018, 12, 9982–9990. DOI: 10.1021/acsnano.8b04265
6. Yoo, J., Yang, K., Cho, B. W., Kim, K. K., Lim, S. C., Lee, S. M., Jeong, M. S. Identifying the Origin of Defect-Induced Raman Mode in WS_2 Monolayers via Density Functional Perturbation Theory. *The Journal of Physical Chemistry C* **2022**, 126, 4182–4187. DOI: 10.1021/acs.jpcc.1c10258.
7. Barbosa, A. do N., Diaz Mendoza, C. A., Stand Figueroa, N. J., Terrones, M., Freire Júnior, F. L. Luminescence enhancement and Raman characterization of defects in WS_2 monolayers treated with low-power N_2 plasma. *Applied Surface Science* **2021**, 535, 147685. DOI: 10.1016/j.apsusc.2020.147685.
8. Han, Z., Wei, T., Xiao, Q., Zhong, X., Xiang, D., Liu, T. Fabrication of patternable Janus transition-metal dichalcogenides assisted by electron beam irradiation. *Appl. Phys. Lett.* **2022**, 120 (22), 221901. DOI: 10.1063/5.0095650.
